# How language skills and working memory capacities explain mathematical learning from preschool to primary school age: Insights from a longitudinal study

**DOI:** 10.1371/journal.pone.0270427

**Published:** 2022-06-24

**Authors:** Nurit Viesel-Nordmeyer, Alexander Röhm, Anja Starke, Ute Ritterfeld

**Affiliations:** 1 Lyon Neuroscientific Research Center (CRNL), University of Lyon, Lyon, France; 2 Department of Rehabilitation Sciences, TU Dortmund University, Dortmund, Germany; 3 Department of Pedagogy and Educational Sciences, University of Bremen, Bremen, Germany; Max-Planck-Institut fur Kognitions- und Neurowissenschaften, GERMANY

## Abstract

Between the age span of 3 to 6 years the foundation for children’s mathematical learning (i.e., numerical abilities and cognition) are laid. However, the developing relations between mathematical skills, language, and working memory starting at preschool age and evolving into primary school age are not well understood. Adopting an empirically validated analysis model, the present study examines in detail longitudinal interdependencies between mathematical skills, a wide range of language skills, and working memory components underlying the mathematical learning process of 41 German preschool children (41.5% female) spanning ages 4 to 6. Phonological processing skills and expressive grammar skills emerged as the most significant language skills for the process of children’s mathematical learning across the investigated age span. Within the latter, children’s phonological processing skills and expressive grammar skills were supported by children’s word expression abilities. The phonological loop emerged as the most important working memory component for children’s early mathematical learning between ages 4 to 6. Furthermore, a wide array of language skills were associated with complex information and storage processes within this mathematical learning process. In conclusion, the present findings provide a more detailed and deeper insight into the learning process of children’s number concept, emphasizing the influence of phonological and particularly grammatical skills.

## Introduction

Understanding the process of early mathematical learning has been the aim of many scholars over the past decades. During recent years, it has become increasingly obvious that children’s mathematical skills are often linked to their language development (e.g., [1, 2]) as well as their working memory capacity (e.g., [3, 4]), or a combination of both (e.g., [5–8]). However, the longitudinal relations between all three components—language skills, working memory and mathematical skills—underlying this process of mathematical learning from preschool to primary school age are still far from being understood. However, within this important age span, which can be set between 3 and 6 years, the foundation for children’s mathematical learning (i.e., numerical abilities and cognition) are laid [9].

According to Dehaene’s [10] *triple-code model for numerical cognition*, numbers and quantities (as the key elements of mathematical learning) are mentally processed as verbal (i.e., language representation; e.g., number words), visual (i.e., symbolic representation; e.g., Arabic digits), and approximate codes (i.e., non-symbolic representation; e.g., number line orientation). Hence, differentiated working memory components, based on the hierarchical model of Baddeley (phonological, visual-spatial and central executive working memory; e.g., [11]), as well as various language skills which are necessary for verbal processing (e.g., lexical and morphological knowledge, phonological processing skills) have been identified as important for children’s early mathematical learning processes (e.g., [3, 12]).

However, little is yet known about which language skills (e.g., phonology, morphology, vocabulary; [13]) are specifically linked to which mathematical skills in children’s early mathematical learning. Furthermore, although the importance of different working memory functions, particularly executive functions or phonological processing, is well documented for specific math (e.g., calculation; [14]) as well as specific language skills (e.g., word learning; [15]), the direction of the relations is not yet fully understood. Based on recent research, a reciprocal and recursive interaction between children’s working memory and their math and language skills is currently discussed [16].

In addition, a recent secondary analysis of data from a large-scale panel study (*n* = 354) [8], in which different parameters of all three domains—language skills, mathematical skills, and working memory—were examined in parallel spanning ages 4 to 8, first points to each domain’s differential importance within the mathematical learning process. Second, the analysis revealed a complex interweaving of the different parameters of all three domains within this process, including reciprocal connections between children’s working memory and their math and language skills. However, the aforementioned panel dataset [8] did not account for a diversity of language skills such as differentiated phonological, morphological, and lexical abilities that seem to play an important role within the mathematical learning process in pre- and primary school age as underlined by a recent meta-analysis (cf. [1]). Additionally, direct measurements of all three working memory components (cf. [11]), which are all important for mathematical learning processes within the aforenamed age span (e.g., [17–19]), were not available within the panel study. Consequently, a finely grained longitudinal perspective on the relation between all three domains using more differentiated and specific language, mathematical, and working memory measures is necessary to engender a better understanding of children’s mathematical learning preceding primary school age. To this end, the present study adopts the analysis model from Viesel-Nordmeyer et al. [8], which has been empirically validated with a large panel sample, and applies it to a much smaller but—with respect to language and working memory—more detailed data set.

To strengthen the line of argumentation the following sections will elaborate on the state of research concerning (1) the role of specific language skills for mathematical learning and (2) working memory as a third variable of the relation between language skills and mathematical learning within the important age span of 3 to 6 years.

### The role of language skills in mathematical learning

In the preschool years—roughly between ages 3 to 6—basic understanding of the numerical concept is acquired (e.g., [20]). This concept is fundamental for academic mathematical learning. Development models concerning early mathematical learning (e.g., [9, 21]) indicate a special role of language in this age span via different functions. First of all, language skills are crucial for the acquisition of the number words and the number-word sequence that children start to learn between ages 2 and 3 (e.g., [9]). The number-word sequence is initially learned purely as a series of connected words or vocabularies, at first without making connections between quantities and numbers (e.g., [22]). Therefore, it comes as no surprise that many studies show a close connection between *vocabulary skills* and mathematical learning, even within early age spans such as between 11 and 55 months (e.g., [18, 23]).

To reach a full understanding of the number concept, a child needs to connect quantities and numbers (e.g., [9]). Therefore, children need to be able to understand and use comparatives or prepositional phrases. Consequently, the use of *grammar skills* alongside vocabulary skills becomes necessary beginning roughly from the age of 3 (e.g., [24]). This has been shown for example in a study by Purpura and Reid [24] which provided evidence for the importance of specific math-related language skills for children’s mathematical learning between the ages of 3 and 5. These math-related language skills are characterized by abilities using prepositions, comparatives and superlatives related to quantities and numbers. However, many studies investigating the relation between language skills and preschool mathematical learning have only taken expressive or receptive vocabulary into account (e.g., [17, 23]). To the authors’ knowledge there are only a few studies in which the importance of specific grammar skills for early mathematical learning has been considered or could be confirmed as relevant factors for mathematical learning (e.g., [6, 25]). From these studies, however, a clear picture regarding the unique explanatory value of both grammar and vocabulary skills for early mathematical ones cannot be obtained. Either a parallel consideration of vocabulary and grammar skills in the studies was not available (e.g., [25]) or both language skills were integrated into one common language construct (e.g., [6]).

In addition to vocabulary and grammar, *phonological processing skills* also play a role in mathematical learning prior to school enrollment (e.g., [26–28]). A comprehensive review of brain imaging studies [26] has reported a strong importance of phonological processing skills (i.e., phonological awareness) for basic mathematical learning. Unfortunately, due to a lack of longitudinal data in the brain imaging studies included in the review, the specific phase within the development of basic mathematical skills where phonological processing skills play the most prominent role could not be exposed [26]. In the few present longitudinal behavioral studies which have considered the influence of phonological awareness on mathematical learning [27, 28], phonological awareness itself was measured at only one time point. Moreover, both behavioral studies revealed heterogeneous results regarding whether pre-school-measured phonological awareness predictes advanced mathematical skills (e.g., [27]) or not (e.g., [28]). Factoring both phonological awareness and vocabulary skills into their analysis, LeFevre et al. [27] could at least demonstrate a unique explanatory value of those language domains—phonological awareness and vocabulary skills—for mathematical learning preceding primary school.

Taken together, the present findings on the co-development of language and mathematical skills prior to school enrollment between the ages of 3 and 6 years underline the need for a more differentiated longitudinal investigation, considering in more detail both language and mathematical skills.

### Working memory as a third variable in the relation between language skills and mathematical learning

Since the storage and retrieval of basic mathematical facts is necessary for the mental processing of numbers [10], the importance of the role of working memory as a third influencing variable becomes obvious [26]. Before focusing on a potential three-way relation between working memory, language skills, and mathematical learning (cf. [26]), the contribution of the working memory system for language and mathematical skills will be briefly discussed. Based on Baddeley’s [11] hierarchical model, working memory is described as an information processing system for learning, roughly divided into three components: the *central executive* as a higher-level component for controlling and coordinating information processing as well as two helping components for short-term storing and maintenance processes of verbal or auditive (*phonological loop*) and visual or spatial (*visuo-spatial sketchpad*) information.

Insights from numerous studies underline the importance of the various components of working memory for language development (e.g., [29, 30]) as well as mathematical learning (e.g., [17–19]). However, the proportional importance of individual working memory components in both domains has not been conclusively clarified. Not only is the phonological loop particularly relevant for language skills like word learning (e.g., [29]), there are also indications of a strong influence by the central executive on grammatical learning and sentence processing [31].

Regarding mathematical learning processes in general, there is evidence for a contribution by all three components (cf. [4]). Yet, heterogeneous results on direct relations between different working memory components and mathematical skills have been found, especially for the important development span between 3 and 6 years. For example, Cornu et al. [17] assessed relations between 141 preschoolers’ verbal and visuospatial abilities and their verbal number skills with differentiated tasks. The authors found that the visuo-spatial sketchpad holds a unique importance for the acquisition of early mathematical skills for 5−6-year-olds. Preßler et al. [32] observed a strong longitudinal relation between both reduced visuo-spatial as well as phonological working memory capacity and weak achievement in early mathematical skills of 92 preschool children at the age between 5 and 6 years. In addition, Röhm et al. [6] examined cross-sectional links between language development, mathematical basic skills, and working memory of 30 kindergarten children at the age of 5 using developmental test batteries. They could detect neither any relation between visuo-spatial sketchpad and early mathematical skills nor any direct relation between the phonological loop and early mathematical skills in children. In their study, however, a direct relation between the central executive and early mathematical skills was shown.

Although, direct comparability and generalizability of the findings is rather limited due to the variability of sample sizes and characteristics (e.g., age or school grade), study designs, measures (e.g., differentiated tasks vs. test batteries), learning contexts (e.g., kindergarten vs. preschool), and control variables (e.g., SES; [19]) among those studies, different explanations for the presented disparate findings can be assumed. As Lee and Bull [19] conclude from their cross-sequential study on children’s working memory, updating, and mathematical performance, first, there are developmental patterns underlying those relations “that vary with grades and the domain of math under consideration” (p. 880) and, second, children’s early learning experiences influence general as well as specific abilities in those domains. On the one hand, the involvement of individual working memory components within mathematical learning could be related to the use and recall of different calculation strategies and existing previous knowledge, particularly depending on children’s individual prerequisites (e.g., [4, 19]). Accordingly, it can be assumed that—in contrast to aided and curriculum-based learning in school—children’s mathematical learning at the age between 4 and 6 is mainly unguided and takes place in engagement with the everyday environment (cf. [6]). Thus, children’s learning success depends even more on their individual cognitive and linguistic competencies at that age. On the other hand, the additional influence of language skills in the development of numerical thinking (cf. [10]) as well as in the communicative and cognitive confrontation with mathematical learning content (e.g., [1]) has so far been neglected in the strand of research focusing on working memory. Looking at the interdependencies of all three domains—language, mathematics, and working memory—in a more differentiated way plus focusing on the age span up to school entry (e.g., [20]) could provide new, valuable insights into the crucial process of mathematical development.

So far, results of four cross-sectional analyses (cf. [5–7, 33]) provide first and important information regarding the three-way relation of interest. In all four studies, no direct relations between the phonological loop and basic mathematical skills were found when language skills were considered. These findings were independent of whether phonological awareness (cf. [7, 33]), expressive and receptive grammar skills (cf. [6]) or expressive vocabulary skills (cf. [5]) were included. Moreover, an additional mediation of the relation between the central executive and early mathematical skills by phonological language skills (cf. [8, 33]) suggests a special role of different language skills within various aspects of information processing. However, the direction of the relation between all three components—language skills, mathematical skills, and working memory—cannot be clarified by the aforementioned cross-sectional analyses. For the sole relation of the two domain-specific areas of language and mathematical skills, a one-way relation in which various language skills directly affect mathematical learning (and not in turn) can be assumed based on longitudinal studies (e.g., [34]). However, the longitudinal relation between one of the domain-specific components (language or mathematical skills) and the domain-general working memory has not yet been clarified. Especially since Hasselhorn and Gold [35] also propose direct and indirect effects of previous knowledge on further knowledge acquisition, it can be assumed that already acquired domain-specific skills can free up working memory resources for more complex mathematical learning. This reasoning is supported by the findings of a recent meta-analysis by Peng, Lin and colleagues [1] which demonstrated that working memory explains an important part of the relation between language and mathematical skills.

More precisely, building on the early development theory of Werner and Kaplan [36], who assumed a spiral pattern of important skills within child development, the idea of a release of capacity within a limited system of working memory facilitated by previous knowledge which then freed up resources for further knowledge building seems justified. For instance, examining the acquisition of vocabulary skills spanning ages 4 to 6, Gathercole and colleagues [30] found a reversing relation pattern between phonological working memory and vocabulary skills when both factors were continuously measured in parallel. Specifically, the results show a strong influence of the phonological loop, measured at age 4, on vocabulary skills measured one year later. However, the reported direction of the relation between phonological working memory and vocabulary skills thereafter lost its significance. Instead, a strong prediction of the performance of the phonological loop at age 6 by existing vocabulary skills at age 5 emerged.

In contrast, a more recent study by Miller-Cotto and Byrnes [37] focused on the relation between children’s central executive working memory and their reading skills from the end of kindergarten until the second grade. The results revealed another picture in which children’s central executive and reading skill performance measured in parallel showed a continuous reciprocal relation. Moreover, the authors found confirmation for a similar mutual relation pattern between the central executive and mathematical skills over the same period [37]. Thus, Hasselhorn and Gold’s [35] theoretical assumption of a direct and indirect effect of previous knowledge on further knowledge acquisition can be supported by recent empirical findings concerning the relations between language, mathematical skills, and working memory up to school age. However, little is known regarding the longitudinal relation pattern between all three domains in parallel.

### Panel-data results of language, mathematical, and working memory skills in parallel

Based on a comprehensive panel data set (National Educational Panel Study; NEPS; [38])) some insights into longitudinal interdependencies between all three domains—language, mathematics, and working memory—for the age span between 4 and 8 years are already available [8]. These include measures of: receptive vocabulary and grammar skills, a comprehensive mathematical construct comparable to PISA (cf. [39]), direct (phonological loop, central executive) and indirect (central executive, visual storage) measurements of working memory, and a large number of background control variables (sex, migration, socioeconomic background, home spoken language, presence of language weaknesses), which could be included in the analyses described below. The results (cf. [8]) of longitudinal path-analyses showed firstly a long-term direct effect of children’s language skills on their mathematical learning, which is more evident for grammar than for vocabulary skills. Secondly, children’s grammar skills in the second year of kindergarten at age 4 to 5 predict the capacity of their phonological and central-executive working memory one year later. Moreover, children’s phonological and central-executive capacities in this age span predict in turn their grammar skills during the first grade at age 6 to 7. Taken together, there seems to be a mutual relation pattern between children’s grammar skills and both working memory components spanning ages 4 to 7, a pattern similar to the one described by Gathercole and colleagues [30] for vocabulary knowledge building including phonological working memory. For the children’s mathematical learning process between 5 and 8 years, an age-dependent involvement of different working memory components could be found [8]. To specify, a direct relation between children’s central executive capacity and their mathematical skills at preschool age was revealed, supported by additional cross-sectional analyses of the panel study. In contrast, as observed in the previous cross-sectional studies, the relation between the phonological loop and mathematical skills seemed to be only indirect, mediated by language parameters [5, 6] and the central executive [6]. However, the longitudinal results uncovered that, conversely, children’s mathematical skills at school age appear to be directly predicted by their phonological working memory and indirectly predicted by their central executive working memory. To gain insights into the longitudinal relation between children’s mathematical skills and working memory capacity, indirect working memory measures (central executive, visual storage, cf. [40]) were used. As far as the NEPS data allowed, a consistent mutual effect between children’s mathematical skills and working memory could be found spanning ages 5 to 8. Finally, the link between children’s preschool grammar skills and their mathematical skills in pre- and primary school age was shown to be mediated by phonological and central executive working memory. In line with Peng, Lin and colleagues [1], the authors argued for a working memory relieving role of language skills that supports mathematical learning up to primary school.

Following the results of the latest meta-analysis conducted by Peng, Lin and colleagues [1], in which correlations concerning all three domains—language, mathematics, working memory—across 101 studies are included, it becomes obvious that there is a need to take a closer look at more differentiated language skills for mathematical learning. Especially necessary would be the consideration of phonological processing (e.g., phonological awareness), as well as receptive (e.g., listening comprehension) and expressive (e.g., word production) language skills in parallel while examining mathematical learning processes. Therefore, due to the strong importance of children’s number concept acquisition for academic mathematical learning (e.g., [20]), the aim is to understand in greater detail the interrelations of language skills, mathematical skills and working memory in the age range of 4 to 6 detected in the panel study [8]. Following other scholars (e.g., [17–19]) who found heterogeneous effects of different working memory components on early mathematical learning in the abovementioned age span, the present study also seeks to adopt a more differentiated direct measurement of this prominent cognitive system (e.g., [11]).

### Research interest

**Fig 1:**
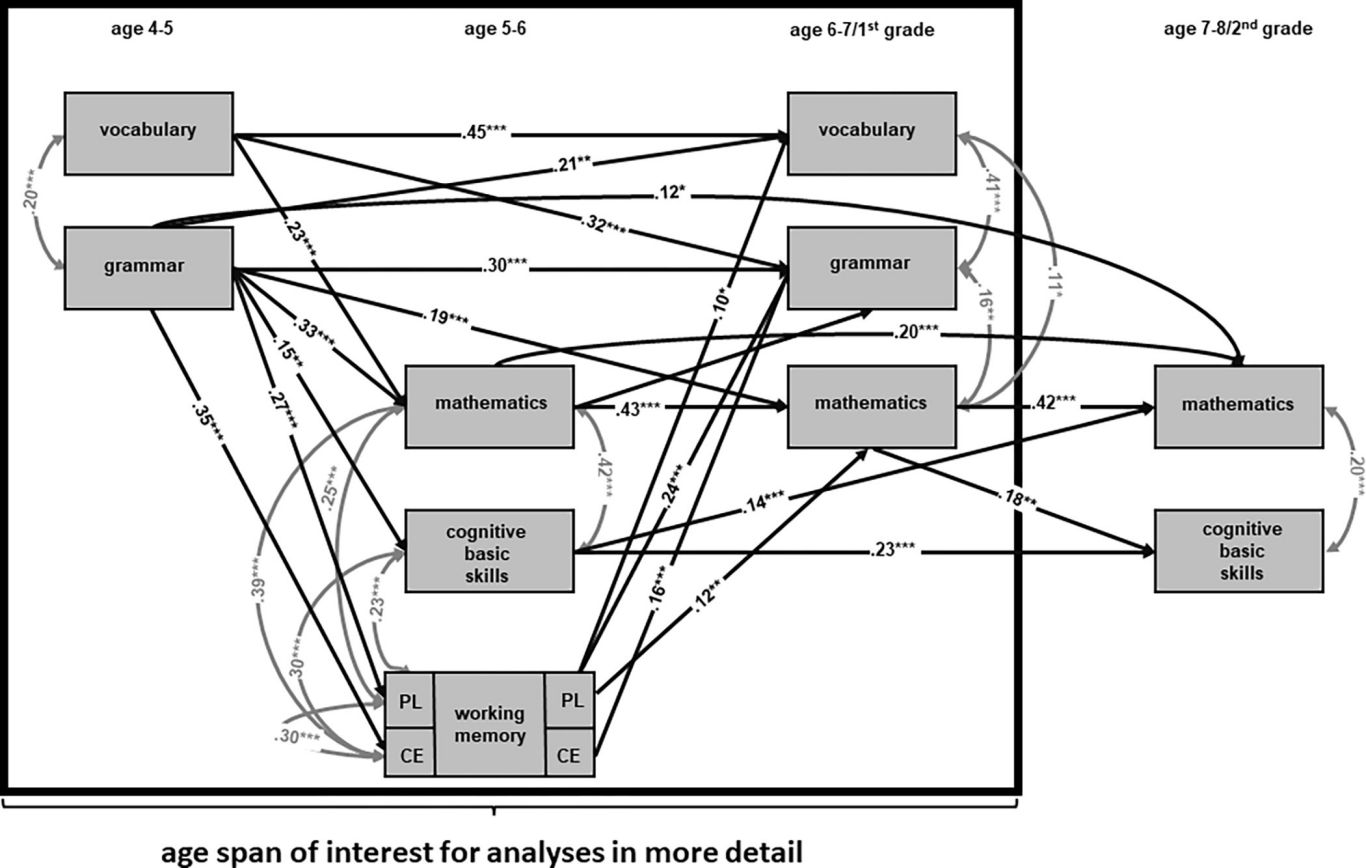
Already existing longitudinal results of the National Educational Panel Study (*n* = 354; see p.139, Journal für Mathematik-Didaktik 2020 [8]) on the interdependencies between language skills, mathematical skills, and different working memory components within the mathematical learning process spanning preschool age until primary school enrollment.

Building on previous work (cf. [8]), the aim of this study is to shed more light on the developmental interdependencies between language skills, mathematical skills, and different working memory components spanning ages 4 to 6 in detail (Fig 1) by using a more elaborated dataset than is available from the NEPS data in order to answer the following research questions:

*Research Question 1 (RQ1)*: Do specific language parameters (phonological awareness, expressive vocabulary, expressive and receptive grammar) predict mathematical skills spanning ages 4 to 6 directly and if so, are there differences in the relation patterns between the differentiated language parameters and mathematical skills within this age span? Based on the results of the few studies which consider the specific role of differentiated language skills on mathematical learning in parallel (e.g., [7, 27]), it can be assumed that the variety of language skills included in this study shows a unique explanatory value in addition to the predominant role of grammar skills.

*Research Question 2 (RQ2)*: Is there a mutual relation pattern between the differentiated language skills and different working memory components (phonological loop, visuo-spatial sketchpad, central executive) within the knowledge building of language skills or between the mathematical skills and these different working memory components within the knowledge building of mathematical skills spanning ages 4 to 6? In accordance with the development theory introduced by Werner and Kaplan [36] and based upon the latest discussion regarding bidirectional relations between cognitive and academic domains [16], a spiraling developmental process over time is presumed. In this regard, the presumption of a mutual relation refers to effect patterns in which, for example, language or mathematical skills at an earlier age are not only affected by working memory, but also have by themselves an effect on later working memory as well as later language and mathematical skills.

**Fig 2:**
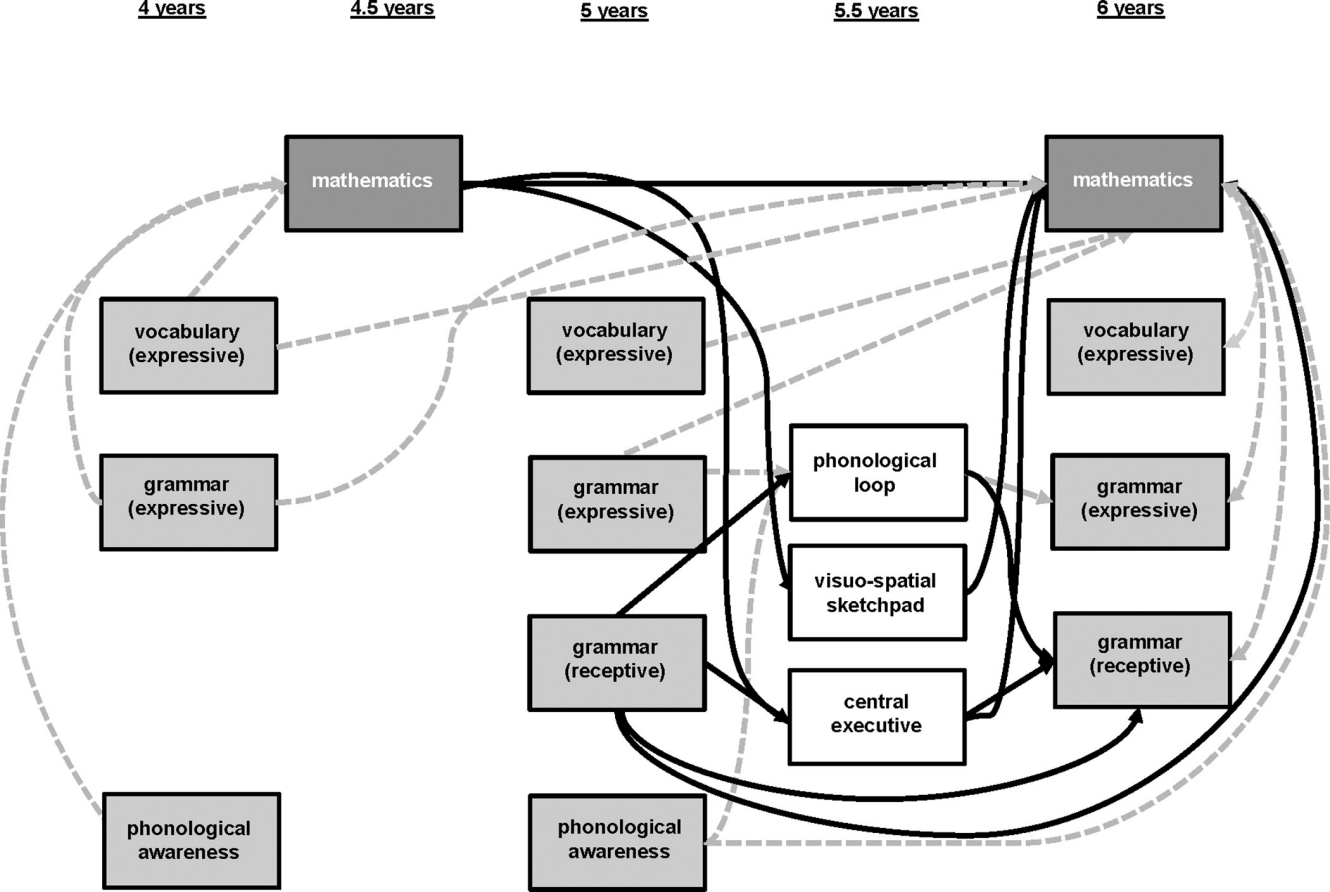
Expected interdependencies between different language skills and math skills as well as different language or math skills and working memory within the acquisition of the number concept. Black errors indicate expected interdependencies based on already existing longitudinal results (cf. Fig 1), grey errors indicate expected interdependencies based on the theoretical background described.

*Research question 3 (RQ3)*: Which role do the proposed mutual relation patterns (language skills and working memory; mathematical skills and working memory) play for the aforementioned relation between differentiated language skills and mathematical skills (RQ1) within the mathematical learning process spanning up to school enrollment? Building on the described results of the large-panel analyses [8], effects of language parameters on mathematical learning mediated by working memory as well as additional working memory effects on mathematical learning mediated by language parameters are assumed (see Fig 2).

## Method

### Design

The present study represents a secondary analysis of a data excerpt from a previous longitudinal study (cf. [41]). Between the ages of 4 and 6 years, children were assessed at five time points (wave 1 (w1): 4.0 years; wave 2 (w2): 4.5 years; wave 3 (w3): 5.0 years; wave 4 (w4): 5.5 years; wave 5 (w5): 6.0 years) on their non-verbal intelligence (wave 1), expressive vocabulary skills (w1, w3, w5), expressive and receptive grammar skills (w1, w3, w5), phonological awareness (w1, w3, w5), basic mathematical skills (w2, w5), and working memory (w4). For further information, see Lüke et al., [41–43].

### Participants

The sample analysed here consisted of *N* = 41 preschool children (17 girls, 24 boys). All children were raised as monolingual German speakers and were recruited via their pediatricians during their regular medical check-up between the ages of 10 and 12 months. Parents were then written informed about the study and the informed consent to participate in this study was provided by the participants’ legal guardian/next of kin. The original study has been reviewed and approved by the Internal Review Board of the University of Münster (2011-517-f-S) and the Internal Review Board of Bielefeld University (EUB 2015–079) (cf. [41–43]). According to the pediatricians as well as standardized results from the developmental test for children aged 6 months to 6 years (ET 6–6; [44]), all children were developing typically. At age 3, 84,5% of the children already attended a German Kindergarten; at age 6, 34,1% of the children were enrolled in primary school. Children’s socioeconomic background (SES) was assessed using the German Socio-Economic Panel (SOEP; [45]) classification of parents and the net equivalent income of the family. The SOEP also takes parental educational attainment and occupational qualifications into account. At age 3.5, children’s non-verbal IQ was measured using the non-verbal IQ-test SON-R 2 ½-7 [46].

The used data stem from a longitudinal study of 45 German children and their primary caregivers (96% mothers) (cf. [41]). The data of four children were excluded because they did not participate after the age of 36 month (*n* = 2) or at the testing with age 5 (*n* = 1) as well as because of a chronic otitis media with several effusions (*n* = 1).

### Instruments

#### Language skills

Children’s expressive vocabulary skills, expressive grammar skills, as well as phonological awareness were assessed at ages 4, 5, and 6 with the subtests vocabulary, grammar, and rhymes from the standardized *Potsdam-Illinois test of psycholinguistic abilities* (P-ITPA; [47]). The subtest *vocabulary* (w1: α = .85; w3: α = .69; w5: α = .80) examines word knowledge and the ability to recognize relationships between words. It tests the extent to which a word can be correctly recognized when only a single attribute of that word is mentioned (e.g., “I’m thinking of something that has fins. What could that be?”). The subtest *grammar* (w1: α = .90; w3: α = .91; w5: α = .90) ascertains how reliably children can already form morphologically coherent connections. For instance, they were asked to complete an incomplete sentence (e.g., “This is a hand. These are two …”) with the support of images. Most tasks require the formation of the plural of nouns, the intensification of adjectives as well as the formation of the preterit and perfect tense but also tap correct usage of the genitive, dative, and accusative. Children’s phonological awareness was measured with the subtest *rhymes* (w1: α = .90; w3: α = .87), in which the children have to decide which of three or four nouns rhymes with a given word. In order to be able to assess phonological awareness largely independently of short-term memory performance, the children are supported by pictures (e.g., "What rhymes with seesaw *(German*: *Wippe)*? Winter, children, vase, shovel *(German*: *Schippe)*?”).

Children’s receptive grammar skills (w3: α = .84; w5: α = .74) at ages 5 and 6 were further measured with the German adaptation of the standardized *test for reception of grammar* (TROG-D; [48]), in which language comprehension is assessed across a total of 21 grammatical areas, including, for example, nouns, negation, prepositions, and plural or passive forms. The understanding of each grammatical structure is tested with the help of four multiple-choice tasks in which a target picture matching an auditory test sentence must be selected from a set of four pictures.

#### Basic mathematical skills

A standardized test for mathematic and arithmetic concepts at preschool age (MARKO-D; [49]) was used to measure basic mathematical skills at ages 4 and 6 (w2: α = .79; w5: α = .85) across five developmental levels with a total of 55 tasks. For level 1 (*counting numbers;* 16 tasks) children are asked, for example, to count as far as possible or divide a quantity into two equal quantities. Concerning level 2 (*ordinal number line;* 10 tasks), tasks include naming the predecessor and successor of a number as well as merging, dividing, modelling, and comparing quantities. Competences for level 3 (*cardinality and decomposability;* 12 tasks) are assessed, for instance, through addition and subtraction of quantities (e.g., 3 plus 3; 5 minus 3) or continuing to count up by one from 7. Level 4 (*containment and class inclusion;* 5 tasks) tests whether a total quantity is recognized as a combination of two partial quantities and the number line is represented as congruent intervals (e.g., 1, 3, 5). For level 5 (*relationality;* 12 tasks) children are asked, for example, to determine the difference between quantities and numbers as well as to count backwards in congruent intervals (e.g., 10, 8, 6).

#### Working memory

In order to map all three components of working memory (cf. [11]), the central executive was measured by the digits backwards task, the phonological loop by the digits span task, and the visuo-spatial sketchpad by the matrix task from the computer-aided standardized *working memory test battery for children from 5 to 12 years* (AGTB 5–12; [50]) at age 5.5. This test battery uses an adaptive algorithm for item selection, scoring, and dropout criteria with the goal of adjusting the requirements depending on children’s age and performance. In the *digits backwards* subtest, sequences of digits (e.g., 7-3-9) with a length of two to seven digits are presented auditorily and must be reproduced correctly by the children in reverse order (e.g., 9-3-7). For the *digits span* subtest sequences of digits similar to the digits backwards tests are presented auditorily and must be reproduced correctly in the same order. The *matrix* subtest presents a 4 × 4 field matrix on which patterns consisting of two to eight black fields are displayed. Immediately afterwards, the children are asked to reconstruct the pattern they have just seen by touching the touchscreen.

### Data analysis

Since this study’s research interest is to acquire a deep insight into the longitudinal interdependencies between all three domains—language skills, mathematical skills and working memory—in parallel, a general model (Fig 2) for computing mediation analyses using the statistics software Mplus 5.21 (cf., [51, 52]) was set up. This model was built upon the assumed interrelations between the three domains of interest, which stem from the already existing longitudinal results (Fig 1) as well as the studies discussed in the theoretical background. Due to the more differentiated measurement of the three domains of interest in this study and this study’s sample size, the ratio of free parameters required a calculation of a separate model for each variable included and its possible predictors from the previous waves (Table 3). For the estimation of direct and indirect effects, bootstrapping with n = 1,000 times as well as the delta method were used (cf., [54]). The scores of the variables used were t-values. Nonverbal IQ (SON-R 2 ½-7; [6]) socioeconomic background, and children’s sex were controlled for in all analyses. For the preceding descriptive analyses (Tables 1 and 2), the statistics software SPSS 27 was used. Descriptive information of all used variables are shown in Table 1.

**Table 1:** Descriptive Analyses of all variables used.

	*N*		*M*	*SE*	*SD*	Skew	*SD* of skew	Kurtosis	*SE* of kurtosis	Min	Max
	valid	absent									
IQ	38	3	76.39	0.68	4.20	-1.77	0.38	3.09	0.75	63	83
Socioeconomic status	37	4	0.01	0.16	0.95	0.53	0.39	-0.09	0.76	-1.76	2.39
Sex	41	0	0.41	0.08	0.50	0.36	0.37	-1.97	0.72	0	1
PhonA (w1)	37	4	52.65	1.37	8.34	0.24	0.39	-0.53	0.76	36	69
Vocabulary (w1)	40	1	52.40	1.53	9.70	0.23	0.37	-0.08	0.73	31	73
Grammar (exp) (w1)	39	2	55.62	1.27	7.93	0.02	0.38	-0.04	0.74	41	76
Math (w2)	40	1	50.68	1.20	7.57	0.11	0.37	-0.99	0.73	39	65
PhonA (w3)	40	1	57.33	1.49	9.39	0.53	0.37	0.18	0.73	40	82
Vocabulary (w3)	41	0	56.93	1.23	7.87	-0.84	0.37	1.05	0.72	32	70
Grammar (exp) (w3)	41	0	57.85	1.58	10.12	0.08	0.37	-0.75	0.72	38	77
Grammar (rec) (w3)	41	0	49.90	1.70	10.89	0.26	0.37	0.23	0.72	29	76
Phonological loop	41	0	50.85	1.87	11.97	-0.18	0.37	-0.91	0.72	29	71
Central executive	40	1	54.80	1.29	8.18	-0.16	0.37	-0.40	0.73	35	71
VSS	41	0	49.88	1.23	7.87	-0.38	0.37	-0.49	0.72	33.00	62.00
Vocabulary (w5)	41	0	55.61	1.38	8.86	-1.15	0.37	2.77	0.72	24	71
Grammar (exp) (w5)	41	0	60.10	1.77	11.33	-0.62	0.37	1.32	0.72	23	78
Grammar (rec) (w5)	41	0	53.29	1.33	8.50	0.44	0.37	0.36	0.72	36	74
Math (w5)	40	1	53.40	1.12	7.07	0.19	0.37	-0.50	0.73	40	68

Note. The different waves are abbreviated with “w”. Grammar (exp) = grammar skills expressive, Grammar (rec) = grammar skills receptive, PhonA = phonological awareness, Math = mathematical skills.

**Table 2:** Bivariate correlations of all used variables.

		(1)	(2)	(3)	(4)	(5)	(6)	(7)	(8)	(9)	(10)	(11)	(12)	(13)	(14)	(15)	(16)	(17)
(1)	IQ																	
(2)	Socioeconomic status	.30																
(3)	Sex	.21	.00															
**Wave 1**																		
(4)	Phonological awareness (w1)	.32	.31	.06														
(5)	Vocabulary (w1)	.09	.49**	.14	.32													
(6)	Grammar (exp) (w1)	.41*	.55**	.29	.38*	.66**												
**Wave 2**																		
(7)	Math (w2)	.51**	.27	.24	.49*	.30	.53**											
**Wave 3**																		
(8)	Phonological awareness (w3)	.18	.55**	.15	.53**	.45**	.55**	.37*										
(9)	Vocabulary (w3)	.08	.50**	.04	.41*	.72**	.56**	.26	.45**									
(10)	Grammar (exp) (w3)	.06	.43*	.14	.45**	.75**	.65**	.25	.44**	.70**								
(11)	Grammar (rec) (w3)	.33*	.47**	.29	.41*	.49**	.60**	.48**	.67**	.37*	.49**							
**Wave 4**																		
(12)	Phonological loop	.37*	.50**	.09	.50**	.62**	.61**	.57**	.43**	.54**	.66**	.57**						
(13)	Central Executive	.56**	.48**	.03	.50**	.15	.44**	.37*	.42**	.23	.34*	.31*	.51**					
(14)	Visuo-spatial sketchpad	.44**	.14	-.06	.20	**-**.06	.04	.31	.04	.07	.04	**-**.06	.32*	.49**				
**Wave 5**																		
(15)	Vocabulary (w5)	.26	.53**	.03	.44*	.66**	.58**	.23	.36*	.80**	.59**	.34*	.62**	.52**	.16			
(16)	Grammar (exp) (w5)	.32	.40*	.04	.39*	.68**	.51**	.46**	.37*	.60**	.76**	.48**	.66**	.29	.08	.64**		
(17)	Grammar (rec) (w5)	.38*	.41*	.23	.36*	.55**	.66**	.39*	.55**	.57**	.39*	.53**	.55**	.33	.10	.59**	.37*	
(18)	Math (w5)	.28	.52**	.23	.56**	.39*	.50**	.58**	.46**	.46**	.63**	.40*	.67**	.70**	.43*	.49**	.51**	.38*

Note. Pearson-Product-Moment correlations; significant values are in bold

*p < .05

**p < .01

***p < .001.

## Results

For a first insight into the relations between the numerous measured language, working memory, and mathematical parameters, Pearson correlations were computed (*p <. 05, **p < .01, ***p <. 001). As can be seen in Table 2, moderate to high values (.39* < *r* < .75**) between all measurements of various language skills within and above the individual measurement time points were found. As expected, the highest correlations could be found between the individual measurement time points of the same construct in language (e.g., vocabulary: .66** *< r* < .72**) and mathematics (*r* = .58**). Aside from expressive vocabulary and grammar at wave 1, all language skills were related with mathematical skills measured at ages 4.5 and 6. In contrast, the individual working memory components showed differentiated relations with mathematical and language skills, as reported below: First of all, with only the exception of their relation to phonological awareness at wave 3 (*r* = .43**), the phonological loop correlated with all language and mathematical skills to a high degree (.50** < *r* < .67**). Relations between the central executive and mathematical measurements were fairly high (.44* < *r* < .67**). For language skills only the central executive and the expressive and receptive grammar skills at wave 1 as well as the central executive and both measurements of phonological awareness were related. In addition, most of the aforementioned variables correlated with the socioeconomic background of the children. In contrast, children’s sex was not related with any of the other measures.

To answer our research questions, it is necessary to gain a deeper insight into the developmental interdependencies between language skills, different working memory components, and mathematical skills within the mathematical learning process up until primary school enrolment. Therefore, results of individual mediation analyses based on a previously established developmental model (Fig 2) dependent on the available data under consideration of background variables (sex, socio-economic background, IQ) were considered.

### Direct influences of language skills on mathematical learning (RQ1)

Concerning research question 1, Table 2 shows that phonological awareness (.34***) as well as expressive grammar skills in wave 1 at the age of 4 (.38***) predict mathematical skills half a year later. Conversely, there is no effect of vocabulary skills on mathematical skills at the aforementioned age point and further to this, no direct effect of language skills on mathematical skills in wave 5 at age 6. Instead, various mediated effects of all measured language skills in wave 1 (age 4) and wave 3 (age 5) on mathematical skills at 6 years were found. These indirect effects will be examined more closely later in relation to the results of research question 3.

### Relation patterns between working memory, language, and mathematical skills within the respective learning processes (RQ2)

Despite the differentiated measurement, working memory was only measured in wave 4 at age 5.5, so results regarding the proposed mutual relation pattern between different working memory components and various language and mathematical skills (RQ2) can only partially refer to the considered development range up to enrollment. For language skills, a mutual relation pattern between expressive grammar skills and especially phonological working memory were found, spanning age 5 to 6 (.34***; .64***). For both other language skills which were measured before and after the different working memory components (expressive vocabulary skills, receptive grammar skills), neither direct nor indirect mutual effects could be found with any working memory component (Table 3). As was detected for the knowledge building process of expressive grammar skills, a mutual relation pattern between mathematical skills and phonological working memory could similarly be found starting already at age 4.5 up to age 6 (.71***;.27***).

**Table 3:** Information regarding direct and indirect effects within the separate models of the general model.

	General effects (direct)				General effects (indirect)				Mediated effects					Fit- indices of separate models						
	*B*	*SD*	*p*	95% CI	*B*	*SD*	*p*	95% CI	Mediator	*B*	*SD*	*p*	95% CI	*Chi* ^*2*^	*p*	*df*	*CFI*	*TLI*	*RMSEA*	*SRMR*
**Wave 1 (4y)/ Wave 2 (4.5y)**																				
Voc w1 → Math w2	−	−	−	−	.16	.09	.07	[.03, .38]	Gram (exp) w1	.16	.09	.07	[.03, .38]	.002	.966	1	1.000	1.163	.000	.001
Gram (exp) w1 → Math w2	.38	.12	< .001	[.13, .60]	−	−	−	−	−	−	−	−	−	.007	.936	1	1.000	1.172	.000	.002
PhonA w1 → Math w2	.34	.10	< .001	[.14, .51]	−	−	−	−	−	−	−	−	−	.000	< .001	1	1.000	1.000	.000	.000
**Wave 1 (4y)/ Wave 3 (5y)**																				
Voc w1→ Voc w3	.51	.12	< .001	[.25, .73]	−	−	−	−	−	−	−	−	−	.000	< .001	1	1.000	1.000	.000	.000
Voc w1 → Gram w3	.70	.09	< .001	[.52, .88]	−	−	−	−	−	−	−	−	−	.030	.862	1	1.000	1.178	.000	.004
Voc w1 → PhonA w3	−	−	−	−	.20	.10	.04	[.06, .45]	Gram (rec) w3	.20	.10	.04	[.06, .45]	.317	.853	1	1.000	1.156	.000	.012
Voc w1 → Gram (rec) w3^a^	.*49*	.*11*	*<* .*001*	*[*.*13*, .*98]*	.26	.15	.08	[.02, .60]	Gram (exp) w1	.26	.15	.08	[.02, .60]	.893	.345	1	1.000	1.016	.000	.024
Gram (exp) w1→ Voc w3	−	−	−	−	.34	.14	.02	[.11, .71]	Voc w1	.34	.14	.02	[.11, .71]	.000	< .001	1	1.000	1.000	.000	.000
Gram (exp) w1 → Gram (exp) w3	.34	.15	.02	[.09, .66]	.43	.16	.01	[.14, .77]	Voc w1	.43	.16	.01	[.14, .77]	1.167	.280	1	.997	.979	.064	.022
Gram (exp) w1 → PhonA^a^ w3	.*65*	.*16*	*<* .*001*	*[*.*34*, *1*.*19]*	.31	.16	.05	[.10, .77]	Gram (rec) w3	.31	.16	.05	[.10, .77]	4.569	.334	4	.994	.971	.059	.039
Gram (exp) w1 → Gram (rec) w3	.47	.22	.03	[-.01, .81]	.36	.15	.01	[.15, .73]	PhonA w3	.36	.15	.01	[.15, .73]	1.002	.606	2	1.000	1.066	.000	.023
PhonA w1→ Voc w3	.31	.13	.02	[.05, .55]	−	−	−	−	−	−	−	−	−	.000	< .001	1	1.000	1.000	.000	.000
PhonA w1 → Gram (rec) w3	.46	.14	.02	[.16, .71]	−	−	−	−	−	−	−	−	−	.000	< .001	0	1.000	1.000	.000	.000
PhonA w1 → PhonA w3	.35	.16	.03	[.04, .62]	.19	.16	.05	[.06, .42]	Gram (rec) w3	.19	.16	.05	[.06, .42]	6.665	.084	3	.953	.781	.17	.068
PhonA w1 → Gram (rec) w3^a^	.*55*	.*19*	.*03*	*[-*.*01*, *1*.*16]*	.10	.09	.26	[-.09, .48]	−	−	−	−	−	.112	.738	1	1.000	1.190	.000	.008
**Wave 1 (4y)/ Wave 4 (5,5y)**																				
Voc w1→ VSS	−	−	−	−	-.04	.15	.81	[-.50, .32]	−	−	−	−	−	55.502	< .001	9	.524	.048	.355	.203
Voc w1 → CentEx	−	−	−	−	.24	.12	.04	[.07, .53]	PhonL	.24	.12	.04	[.07, .53]	7.305	.007	1	.851	.253	.392	.148
Voc w1 → PhonL^a^	.*73*	.*12*	*<* .*001*	*[*.*36*, *1*.*03]*	.42	.17	.02	[.14, .82]	Gram (exp) w3	.42	.17	.02	[.14, .82]	.646	.886	3	1.000	1.104	.000	.033
Gram (exp) w1→ VSS	−	−	−	−	.04	.16	.82	[-.37, .52]	Gram (exp) w3 → PhonL	.26	.12	.03	[.01, .67]	6.64	.249	5	.977	.935	.089	.065
Gram (exp) w1 → CentEx	−	−	−	−	.20	.12	.08	[.04, .50]	PhonL	.20	.12	.08	[.04, .50]	5.252	.154	3	.956	.868	.135	.065
Gram (exp) w1 → PhonL^a^	.*82*	.*17*	*<* .*001*	*[*.*30*, *1*.*28]*	.46	.19	.02	[.06, 1.06]	Voc w1	.46	.19	.02	[.06, 1.06]	4.762	.190	3	.970	.911	.120	.065
PhonA w1→ VSS	−	−	−	−	-.02	.06	.74	[-.19, .15]	Gram (exp) w3 → PhonL	.14	.07	.042	[.01, .37]	12.637	.082	7	.887	.774	.140	.101
PhonA w1 → CentEx	−	−	−	−	.13	.07	.04	[.03, .30]	PhonL	.13	.07	.04	[.03, .30]	2.826	.093	1	.946	.728	.211	.074
PhonA w1 → PhonL^a^	.*64*	.*16*	*<* .*001*	*[*.*18*, *1*.*03]*	.28	.11	.01	[.09, .51]	Gram (exp) w3	.28	.11	.01	[.09, .51]	2.520	.284	2	.987	.955	.080	.055
**Wave 1 (4y)/ Wave 5 (6y)**																				
Voc w1→ Voc w5	−	−	−	−	.38	.17	.02	[.15, .80]	Voc w3	.38	.17	.02	[.15, .80]	.308	.959	3	1.000	1.114	.000	.026
Voc w1 → Gram (exp) w5	.45	.18	.01	[.09, .69]	.44	.16	.01	[.13, .62]	Gram (exp) w3	.44	.16	.01	[.13, .62]	3.186	.527	4	1.000	1.025	.000	.047
Voc w1 → Math w5	−	−	−	−	.32	.09	< .001	[.17, .51]	PhonL	.32	.08	< .001	[.17, .51]	4.540	.338	4	.989	.976	.057	.072
Voc w1 → Gram (rec) w5	−	−	−	−	.34	.11	< .001	[.14, .58]	Gram (exp) w1	.34	.11	< .001	[.14, .58]	3.742	.442	4	1.000	1.010	.000	.059
Gram (exp) w1→ Voc w5	−	−	−	−	.55	.21	< .001	[.23, 1.04]	Voc w1→ Voc w3	.32	.15	.04	[.11, .73]	8.780	.186	6	.972	.934	.106	.074
Gram (exp) w1 → Gram (exp) w5	−	−	−	−	.85	.23	< .001	[.50, 1.43]	Voc w1	.31	.15	.03	[.10, .75]	6.393	.172	4	.975	.925	.121	.095
									Gram (exp) w3	.23	.12	.06	[.05, .53]							
									Voc w1/ Gram (exp) w3	.31	.15	.03	[.10, .70]							
Gram (exp) w1 → Math w5	−	−	−	−	.31	.12	.01	[.13, .61]	PhonL	.31	.12	.01	[.13, .61]	4.274	.370	4	.995	.988	.041	.072
Gram (exp) w1 → Gram (rec) w5	.66	.21	< .001	[.25, 1.07]	−	−	−	−	−	−	−	−	−	.000	< .001	0	1.000	1.000	.000	.000
PhonA w1→ Voc w5	−	−	−	−	.34	.16	.04	[.12, .77]	Voc w3	.34	.16	.04	[.12, .77]	.000	< .001	0	1.000	1.000	.000	.000
PhonA w1 → Gram (exp) w5	−	−	−	−	.41	.16	.01	[.14, .75]	Gram (exp) w3	.41	.16	.01	[.14, .75]	.396	.529	1	1.000	1.080	.000	.039
PhonA w1 → Math w5	−	−	−	−	.20	.07	< .001	[.09, .37]	PhonL	.20	.07	< .001	[.09, .37]	9.999	.040	4	.860	.685	.191	.129
PhonA w1 → Gram (rec) w5	−	−	−	−	.20	.11	.07	[-.05, .38]	−	−	−	−	−	.883	.347	1	1.000	1.022	.000	.032
**Wave 2 (4.5y/ Wave 4 (5.5y)**																				
Math w2 → VSS	−	−	−	−	.14	.09	.12	[-.06, .40]	−	−	−	−	−	4.891	.180	2	.939	.818	.124	.073
Math w2 → CentEx	−	−	−	−	.22	.13	.09	[.03, .53]	PhonL	.22	.13	.09	[.03, .53]	.713	.398	1	1.000	1.045	.000	.037
Math w2 → PhonL	.71	.15	< .001	[.44, 1.00]	−	−	−	−	−	−	−	−	−	6.640	.156	4	.951	.891	.127	.068
**Wave 2 (4.5y)/ Wave 5 (6y)**																				
Math w2 → Math w5	.31	.14	.03	[.02, .55]	.29	.10	.01	[.13, .54]	PhonL	.29	.10	.01	[.13, .54]	.741	.691	2	1.000	1.081	.000	.032
**Wave 3 (5y)/ Wave 4 (5.5y)**																				
Voc w3→ VSS	−	−	−	−	-.07	.16	.64	[-.51, .38]	−	−	−	−	−	1.146	.766	3	1.000	1.104	.000	.028
Voc w3 → CentEx	−	−	−	−	.12	.11	.28	[-.12, .51]	−	−	−	−	−	14.765	< .001	2	.852	-.033	.395	.077
Voc w3 → PhonL^a^	.*81*	.*16*	*<* .*001*	*[*.*34*, *1*.*30]*	.57	.20	< .001	[.24, 1.01]	Gram (exp) w3	.57	.20	< .001	[24, 1.01]	.701	.704	2	1.000	1.097	.000	.026
Gram (exp) w3→ VSS	−	−	−	−	.29	.11	.01	[.08, .68]	−	−	−	−	−	10.885	.054	5	.908	.779	.169	.099
Gram (exp) w3 → CentEx	.29	.12	.01	[-.05, .64]	.00	.00	< .001	[.00, .00]	−	−	−	−	−	.621	.431	1	1.000	1.038	.000	.030
Gram (exp) w3 → PhonL	.34	.12	< .001	[.42, 1.07]	.05	.05	.31	[-.06, .21]	−	−	−	−	−	.000	< .001	0	1.000	1.000	.000	.000
Gram (rec) w3 → VSS	−	−	−	−	.11	.08	.17	[.00, .47]	−	−	−	−	−	8.980	.062	4	.928	.785	.174	.114
Gram (rec) w3 → CentEx	−	−	−	−	−	−	−	−	−	−	−	−	−	16.085	< .001	1	.754	-1.218	.607	.095
Gram (rec) w3 → PhonL	−	−	−	−	.29	.09	< .001	[.14, .58]	Gram (exp) w3	.29	.09	< .001	[.14, .58]	.000	< .001	0	1.000	1.000	.000	.000
PhonA w3 → VSS	−	−	−	−	.16	.07	.04	[.01, .41]	CentEx	.16	.07	.04	[.01, .41]	1.627	.202	1	.980	.900	.124	.040
PhonA w3 → CentEx	.28	.12	< .001	[.04, .50]	−	−	−	−	−	−	−	−	−	7.485	.024	2	.831	.578	.259	.124
PhonA w3 → PhonL^a^	.*48*	.*14*	*<* .*001*	*[*.*05*, .*84]*	.36	.12	.01	[.06, .70]	−	−	−	−	−	.000	< .001	0	1.000	1.000	.000	.000
**Wave 3 (5y)/ Wave 5 (6y)**																				
Voc w3 → Voc w5	.95	.14	< .001	[.63, 1.16]	−	−	−	−	−	−	−	−	−	.740	.691	2	1.000	1.058	.000	.025
Voc w3 → Math w5	−	−	−	−	.31	.11	< .001	[.13, .56]	PhonL	.31	.11	< .001	[.13, .56]	3.598	.308	3	.987	.960	.070	.070
Voc w3 → Gram (rec) w5	−	−	−	−										3.679	.451	4	1.000	1.012	.000	.062
Voc w3 → Gram (exp) w5	−	−	−	−	.64	.19	< .001	[.31, 1.06]	Gram (exp) w3	.64	.19	< .001	[.31, 1.06]	.464	.793	2	1.000	1.078	.000	.017
Gram (exp) w3 → Voc w5	−	−	−	−	.49	.15	< .001	[.25, .81]	Voc w3	.49	.15	< .001	[.04, .33]	3.391	.495	4	1.000	1.021	.000	.061
Gram (exp) w3 → Math w5	−	−	−	−	.28	.09	< .001	[.12, .49]	PhonL	.15	.07	.04	[.03, .27]	3.952	.683	6	1.000	1.054	.000	.056
									CentEx	.14	.06	.02	[.11, .64]							
Gram (exp) w3 → Gram (rec) w5					.49	.16	.002	[.13, 1.00]	Voc w3 →Voc w5	.25	.09	.01	[.07, .53]	8.148	.614	10	1.000	1.038	.000	.064
Gram (exp) w3 → Gram (exp) w5	.69	.17	< .001	[.33, 1.00]	−	−	−	−	−	−	−	−	−	2.698	.441	3	1.000	1.013	.000	.049
Gram (rec) w3 → Voc w5	−	−	−	−	.26	.12	.03	[.01, .70]	Voc w3	.26	.12	.03	[.01, .70]	4.985	.083	2	.938	.815	.191	.116
Gram (rec) w3 → Math w5	−	−	−	−	.20	.07	.002	[.07, .43]	Gram (exp) w3 →PhonL	.11	.05	.02	[.02, .2]	10.019	.124	6	.945	.871	.128	.079
Gram (rec) w3 → Gram (rec) w5	.47	.14	< .001	[.20, .79]	−	−	−	−	−	−	−	−	−	1.310	.252	1	.983	.949	.087	.051
Gram (rec) w3 → Gram (exp) w5	−	−	−	−	.38	.18	.04	[.10, .78]	Gram (exp) w3	.38	.18	.04	[.10, .78]	5.573	.134	3	.952	.856	.145	.076
PhonA w3 → Voc w5	−	−	−	−	.36	.13	< .001	[.17, .67]	Voc w3	.36	.13	< .001	[.17, .67]	.000	< .001	0	1.000	1.000	.000	.000
PhonA w3 → Math w5	−	−	−	−	.27	.08	< .001	[.13, .45]	PhonL	.14	.06	.01	[.05, .27]	90.862	< .001	21	.457	.301	.285	.334
									CentEx	.13	.06	.03	[.03, .26]							
PhonA w3 → Gram (rec) w5	.39	.14	.004	[.05, .75]	.16	.07	.01	[.01, .37]	Voc w5	.16	.07	.01	[.01, .37]	.351	.839	2	1.000	1.082	.000	.020
PhonA w3 → Gram (exp) w5	−	−	−	−	.39	.14	.005	[.05, .70]	Gram (exp) w3	.30	.12	.02	[.05, .79]	.000	< .001	0	1.000	1.000	.000	.000
**Wave 4 (5,5y)/ Wave 5 (6y)**																				
VSS → Voc w5	−	−	−	−	.24	.17	.15	[-.07, .79]	−	−	−	−	−	.515	.423	1	1.000	1.094	.000	.021
VSS → Math w5	−	−	−	−	.39	.11	< .001	[.06, .54]	PhonL	.13	.06	.04	[.03, .29]	18.860	< .001	4	.752	.442	.301	.206
									CentEx	.26	.08	< .001	[.11, .45]							
VSS → Gram (rec) w5	−	−	−	−	.24	.11	.03	[.06, .54]	PhonL	.24	.11	.03	[.06, .54]	7.713	.021	2	.788	.363	.264	.133
VSS → Gram (exp) w5	−	−	−	−	.31	.16	.05	[.05, .69]	PhonL	.31	.16	.05	[.05, .69]	2.796	.095	1	.947	.683	.209	.092
CentEx→ Voc w5	−	−	−	−	.31	.14	.03	[.12, .65]	PhonL→ Gram (rec) w5	.13	.06	.03	[.04, .29]	5.455	.605	7	1.000	1.039	.000	.087
CentEx → Math w5	.47	.08	< .001	[.29, .61]	.21	.07	.01	[.09, .37]	PhonL	.21	.07	.01	[.09, .37]	.446	.800	2	1.000	1.082	.000	.022
CentEx → Gram (rec) w5	−	−	−	−	.34	.14	.01	[.11, .64]	PhonL	.34	.14	.01	[.11, .64]	.382	.537	1	1.000	1.101	.000	.025
CentEx → Gram (exp) w5	−	−	−	−	.47	.17	.01	[.19, .87]	PhonL	.47	.17	.01	[.19, .87]	.411	.522	1	1.000	1.082	.000	.026
PhonL → Voc w5	.35	.16	.03	[-.05, .82]	.16	.08	.05	[.01, .46]	Gram (rec) w5	.16	.08	.05	[.01, .46]	10.543	.104	6	.932	.841	.138	.155
PhonL → Math w5	.27	.06	< .001	[.15, .39]	.12	.06	.01	[.04, .24]	CentEx	.12	.06	.01	[.04, .24]	2.301	.316	2	.992	.977	.061	.062
PhonL → Gram (rec) w5	.42	.12	< .001	[.18, .67]	−	−	−	−	−	−	−	−	−	.000	< .001	0	1.000	1.000	.000	.000
PhonL → Gram (exp) w5	.64	.12	< .001	[.29, .85]	−	−	−	−	−	−	−	−	−	10.182	.017	3	.732	.554	.245	.194

Note. Bootstrapping = 1000 as well as the delta method were used for the estimation of direct and indirect effects (cf. Muthén & Muthén, 2017). A missing direct or indirect effect was marked using “−”. The different waves are abbreviated using “w”. Voc = vocabulary skills, Gram (exp) = grammar skills expressive, Gram (rec) = grammar skills receptive, PhonA = phonological awareness, Math = mathematical skills, VSS = visuo-spatial working memory skills, CentEx = central executive working memory skills, PhonL = phonological working memory skills.

### Interdependencies between language skills, mathematical skills and working memory within the mathematical learning process (RQ3)

Aforementioned indirect effects of language skills in wave 1 at ages 4 and in wave 3 at age 5 on mathematical skills in wave 5 at age 6 were mediated by working memory, though, more precisely, almost exclusively by the phonological loop. Considering the addressed language skills in more detail (Table 3), an indirect effect on mathematical skills at age 6 by the phonological loop could be found for expressive vocabulary skills (at age 4 (w1): .32***; at age 5(w3): .31***), expressive grammar skills (at age 4 (w1) .31***, at age 5 (w3): .15**), receptive grammar skills (at age 5(w3): .11***), and phonological awareness (at age 4(w1): .20***, at age 5(w3): .14***). In addition, an effect of expressive grammar skills and phonological awareness at age 5 on mathematical skills at age 6 mediated by the central executive could be found (expressive grammar skills: .14**; phonological awareness: .13**). Since there was no adequate model fit for the effect of phonological awareness at age 5 on mathematical skills at age 6 (see Table 3), the latter result should be interpreted with caution.

Finally, individual direct and indirect effects of the different working memory components on language and mathematical skills in wave 5 at age 6 need to be considered in order to support a better understanding of the importance of different working memory processes during preschool age for language and mathematical performance at school entry. As can be seen in Table 3, the phonological loop (.27***)—as already mentioned—but especially the central executive (.47**) predict mathematical skills at age 6. However, both the phonological loop as well as the central executive each also mediate in part the effect of the other on mathematical performance (Table 3). In addition, an indirect effect of the visuo-spatial sketchpad on mathematical skills at age 6 was also mediated by both phonological (.13*) and central executive (.26***) working memory. With the knowledge that language skills at primary school age play a very special role in advanced mathematical learning, the influence of different working memory components on language skills at age 6 should also be considered. Further to the direct effect of the phonological loop on expressive grammar skills at age 6 (.64***) already reported in connection with the mutual relation patterns above, a direct effect by the phonological loop on receptive grammar skills (.42***) could also be found. In contrast, neither a direct nor an indirect effect of the phonological loop on vocabulary skills at age 6 could be established. Moreover, additional effects of the central executive as well as the visuo-spatial sketchpad on all language skills at age 6 have been mediated by phonological working memory performance. To conclude, running the reported mediation analyses demonstrated a large number of direct and indirect relations between the differentiated language skills—phonological awareness, expressive vocabulary skills, expressive and receptive grammar skills—within the longitudinal development process spanning ages 4 to 6 (for more detail see Table 3).

## Discussion

Building on recent research, the present study sought to clarify open questions regarding specific interdependencies of language skills, mathematical skills, and different working memory components underlying the crucial learning process of the number concept between the ages of 4 and 6 years. To do so, and using an already empirically validated analysis model [8], various language skills were considered in parallel at short time interval measures in addition to a comprehensive working memory construct and mathematical skills spanning ages 4 to 6. Despite the comparably small sample size, the present findings provide a more detailed and deeper insight into the learning process of the number concept within the context of established models [10] and developmental theories [9, 21] for mathematical learning and cognition.

### The role of specific language skills in early mathematical learning

In line with findings from a recent meta-analysis [1], phonological processing skills as well as receptive and expressive vocabulary and grammar skills were considered. This parallel and longitudinal measurement of numerous language parameters made it possible to address the open question (cf. [26]) of when phonological processing skills play the most prominent role in the acquisition of basic mathematical skills. As mentioned previously in the theoretical section, the importance of phonological processing skills—in particular phonological awareness—for the acquisition of basic mathematical skills has already been demonstrated in several brain imaging studies but a longitudinal consideration has so far been lacking (cf. [26]). The findings of this study with repeated measurements of phonological awareness, however, do just underline the results of two longitudinal studies with only one phonological awareness measurement included (cf. [7, 28]). More precisely, the findings show that phonological awareness seems to play a role in mathematical skills only at the age of 4. Consequently, the questioned prominent role of phonological processing skills (cf. [26]) within the acquisition of basic mathematical skills seems to be placed in the mathematical development period before children reach a full understanding of the number concept (cf. [28]). Thus, the assumption that language skills support, in particular, advanced mathematical skills by freeing up basic mathematical knowledge from memory (e.g., [1]) cannot be automatically applied to phonological processing skills, which could be explained in different ways: Firstly, the specific phonological awareness measurement in this study using rhymes measured more basic phonological awareness skills (cf. [53]) which presumably become less relevant for phonological processing for advanced mathematical learning during primary school age. Secondly, the importance of phonological processing skills for mathematical learning during primary school age could be overtaken in general by more advanced linguistic skills (e.g., advanced grammar skills) which are more necessary during this period.

Aside from providing answers about the special role of phonological processing skills for mathematical learning, the parallel and longitudinal measurement of various language skills in this study helped to identify the role of vocabulary and grammar skills within the early mathematical learning process up to primary school age. As already shown in the preceding analyses of the longitudinal NEPS data [8], the results of the present study once again highlight the prominent role of grammar skills for preschool mathematical learning in comparison to other language skills spanning ages 4 to 6. However, the parallel examination of the influence of expressive grammar and vocabulary skills on mathematical learning did not yield a direct effect of vocabulary skills on mathematical skills. This is in contrast to the findings from the NEPS analyses reported above [8], in which only receptive language measurements are included, showing a less explanatory value of vocabulary skills for mathematical learning still remains in addition to a strong explanatory value of specifically receptive grammar skills. Thus, it can be assumed that pure ‘general’ lexical skills are not sufficient to develop a deeper understanding of quantities and numbers. Rather, grammar skills like understanding and using prepositional phrases or comparatives seem to be necessary in order to be able to relate quantities to one another. Such grammar skills are also included within the so-called “mathematical language construct” used in different studies (e.g., [24, 54]) and have already been identified as particularly important for the mathematical development period investigated here (e.g., [24]). Nevertheless, Purpura and Reid [24] have also observed that a previously existing influence of vocabulary skills on mathematical learning during preschool age also seems to diminish when measurements for the specific mathematical language construct, including grammar, are considered. The existing vocabulary skills in this study, however, seem to have at least an indirect function within the pre-school mathematical learning process by supporting the grammar skills necessary to communicate. All in all, there is evidence that previous lexical knowledge forms the foundation for mastering the grammar skills necessary to adequately form the number concept.

### Working memory as a third variable in the relation between language skills and mathematical learning

The additional consideration of the third component of interest, working memory, points out one further function of language within the mathematical learning process also highlighted in the latest meta-analyses by Peng, Lin and colleagues [1]. In line with the “thinking function hypothesis” (cf. [1]), a third variable like working memory (cf. [26]) underlies the use of language skills for more complex mathematical learning processes (cf. [1]). The results of this study showed a supporting effect of various language skills at age 5 on both working memory processes—the phonological loop and the central executive—with direct effects on mathematical learning at age 6. However, there were some differences between the importance of the specific language skills for only phonological storage processes (i.e., for the phonological loop) or for even more complex information processing (i.e., the central executive) which included the exchange with long-term stored knowledge. Firstly, phonological awareness skills seem to lighten the load on the phonological loop but also the central executive for processes of mathematical learning. This aligns with the assumption of working memory as one possible explanation for the relation between phonological processing and basic mathematical skills based on the results of brain imaging studies (cf. [26]). Moreover, this finding extends previous attempts to address the prominent role of phonological skills for basic mathematical skills to an additional supporting role of phonological skills for (a) important cognitive storage processes, and (b) more complex information processing within mathematical learning up to the age of 6. Secondly, both expressive and receptive grammar skills at age 5 seem to be helpful for successful cognitive processes of mathematical learning one year later. To be precise, the expressive grammar skills seem to support both phonological and central executive processes of later mathematical learning. Receptive grammar skills, however, seem to only promote the phonological storage processes which are necessary for mathematical learning. These results are in contrast with the findings of the NEPS analyses [8], in which only receptive language measures were included, showing a supportive effect of grammar skills on both phonological and central executive working memory processes but no effect of vocabulary skills. A possible explanation for the heterogeneity in the results of both studies, apart from the more differentiated consideration of language measures here, could also be the different measures of the three components of interest: language, mathematics, and working memory. Beyond this, the results underline a stronger memory-relieving function of expressive than receptive language parameters. This highlights again the seemingly stronger importance of expressive skills to reach a full understanding of numbers. Surprisingly, the expressive vocabulary skills that have so far played a minor role within the mathematical learning process in this study, play a much larger role in mathematical skills at age 6 by supporting phonological storage processes, in comparison to all other language skills. A possible explanation could be the increasing importance of the knowledge of specific mathematical vocabularies for mathematical learning processes during primary school age [55]. Similar to the present findings, results from a previous study of Schmitt and colleagues (e.g., [56]) have even demonstrated a supporting effect by vocabulary skills on the development of cognitive processing, specifically different central executive skills, within the same age span. The results of Schmitt and colleagues [56] show, however, that a parallel measurement of the aforementioned specific mathematical language skills (cf. [24]), aside from general vocabulary skills, seem to be advisable to detect the actual effect of vocabulary skills for the release of working memory. At least for the construct of executive functions [56], the effect of vocabulary skills on cognitive processing skills was lost following the parallel inclusion of specific mathematical language. However, due to that study’s design [56], information could not be given about changes in potentially existing indirect effects on mathematical learning by the cognitive processing skills.

Finally, based on various cross-sectional results from recent studies (cf. [5, 6, 7, 33]) and also some small longitudinal effects within the NEPS analyses [7], a mediation of the working memory’s effect on mathematical learning over language skills was expected. However, the analyses did not yield any mediated effects of the differentiated working memory measurements at age 5.5 on mathematical learning over the language parameters measured at age 6. One of the reasons, as mentioned earlier, may be the measurement of different constructs between the individual studies. The content of the mathematical measurement construct used seems to be particularly crucial for results regarding which specific language skills (e.g., [1]) or specific working memory components (e.g., [4]) are detected as important for mathematical learning.

Returning to this study’s findings concerning the contribution of the different working memory components within the mathematical learning process, more information should be given. As already reported, a direct effect of the phonological loop and the central executive on mathematical learning at the beginning of school age emerged. However, for the visuo-spatial sketchpad no effect has been found. A look at previous studies which examined the role of different working memory components for mathematical skills for this age span also paints a heterogeneous picture, especially regarding the explanatory value of the visuo-spatial sketchpad. Unfortunately, since only a few studies included all three components of working memory, it is difficult to ascertain clear comparability. Furthermore, the results of the remaining studies (e.g., [12, 57, 58]) show all possible variations, making it hard to achieve a clear explanation for the results found here. Evidence of a direct effect of the phonological loop and the central executive on mathematical skills at the beginning of school age could also be found in two studies that included multiple measures of all three components of working memory [12, 57]. Our results regarding the predominant importance of phonological and central executive working memory processes for mathematical skills at the beginning of school age can thus be supported.

### The proposed mutual relations between working memory, language and mathematical skills

There were some unexpected results when investigating the direction of the relation between working memory and mathematical skills within the mathematical learning process. In line with the current discussion (cf. [16]), a mutual relation could be found between working memory and mathematical skills spanning ages 4 to 6. However, in contrast to the results of two larger panel studies (ECLS; cf. [37]; NEPS; cf. [8]) which have shown a mutual relation between the performance of central executive working memory and mathematical skills, our study emphasizes instead the role of phonological working memory. A deeper examination of the instruments used found that in the ECLS and NEPS studies a more comprehensive mathematical construct was used which goes beyond a pure understanding of the numbers concept. In this study, however, a specific measure was deliberately used in order to uncover these basic mathematical skills which precede mathematical learning in primary school. Concretely, abilities such as number words and the number word sequence were included. These abilities were further tapped by the instrument used to measure the phonological loop. Therefore, a mutual relation is expected between the phonological loop (measured by the digit-span task) and mathematical skills (based on a construct that assessed the number word concept). What is more, based on development models of early mathematical acquisition (e.g., [9, 21]), abilities regarding number words and the number word sequence particularly depend on language skills. Conclusively, a clear contrast between all three components—language, mathematics and working memory—should be questioned depending on the instrument used. Finally, in line with the results from the preceding NEPS analyses (cf. [8]), the mutual relation between language skills and the phonological loop could be replicated. This has already been observed by Gathercole and colleagues [30] within vocabulary knowledge building but only for grammar skills. In contrast to the NEPS analyses, the mutual relation pattern was only replicated for grammar skills measured expressively. A possible explanation could be that the rehearsal process of the phonological loop in the sense of an inner speech [59] does not seem to be so pronounced at this age yet according to current knowledge (e.g., [60]). It is possible that skills for inner speech can already support the pure storage process of the phonological loop and thus further promote the knowledge building process of expressive grammar skills. This result also strongly emphasizes the greater importance of expressive language measures detected by the approach of measuring differentiated language skills used in this study. This could possibly originate from a high importance of communicative processes for learning within this age range in which reading and writing skills are still in the background.

### Limitations

By attempting to map the developmental relations between the three components of interest—language, mathematics and working memory—in parallel to as differentiated a degree as possible, a large number of free parameters were automatically given. Based on the available data size (*n* = 41), it was necessary to calculate separate individual models to entail a careful consideration of effect sizes. In addition, for economic reasons, a differentiated survey of all three components considered here was not possible for each measurement time point. Due to the possible confounds discussed concerning all three components, this would have helped to more clearly define the differences between these three components. Moreover, a multidimensional test of mathematical abilities, in addition to the differentiated test of language abilities and working memory, is lacking, which would have been helpful to uncover even more specific interdependencies between all three components of interest. In order to adapt the longitudinal development model of language, mathematics, and working memory from the previous, bigger panel study [8], the decision had been made to also include the same measures for the phonological loop as well as the central executive in this study. However, the digit span forward test for the phonological loop as well as the digit span backward test for the central executive are numeric and therefore have been discussed as automatically related to numerical skills. Ultimately, it is recommended that different instruments be used for the same parameter in each case when taking up the discussed simultaneous and differentiated measurement of all three components of interest in further research.

Concerning the observed mutual relations between language and mathematical skills as well as working memory, findings must be interpreted with caution due to the one-time measure of working memory at age 5.5. Hence, it cannot be fully ruled out that the found link between children’s mathematical skills at age 4.5 and, for example, the phonological loop at age 5.5 did occur because of an already present relation between both domains at age 4.5. Future studies should aim to adopt measures for all domains at multiple timepoints.

Finally, there are few separate models in which an adequate model fit could not be reached. This applies in particular to models in which the visuo-spatial-sketchpad has been incorporated. To address this problem, a more comprehensive measurement of the visuo-spatial sketchpad with a clear distance to the nonverbal IQ test should be used in further studies with a larger sample size.

## Conclusion

In summary, the findings contribute towards a deeper understanding of the interdependencies between language skills, mathematical skills, and working memory within the acquisition of the number concept preceding mathematical skills at primary school age. Simultaneous consideration of differentiated language measures in the mathematical learning process up to primary school enrollment particularly highlighted a special role played by phonological processing skills as well as expressive grammar abilities, supported by word production abilities, in reaching a full understanding of numbers. In addition, comprehensive language skills during the preschool age range seem to be necessary for supporting cognitive skills which serve as basis for the mathematical learning process [cf., 56]. More specifically, cognitive skills of phonological storage (from phonological loop) and more complex information processing (from central executive working memory) seems to underly the successful acquisition of mathematical skills which are necessary for advanced mathematical learning processes in school age. Moreover, our analyses especially emphasized the role of expressive language skills, which underlines the importance of interaction and communication within the process of reaching a full understanding of numbers. Children’s home learning environment, which has so far been neglected for mathematical acquisition, should also be given a strong focus in future work. Based on initial studies and supported by the present findings, it can be assumed that potential communicative and interactive processes with numerical content within this early learning environment offer ideal preparation for the starting conditions of learning advanced mathematics at primary school age (cf. [61]).

## Supporting information

S1 Data (SAV)
